# Discovery of Bioactive Metabolites in Biofuel Microalgae That Offer Protection against Predatory Bacteria

**DOI:** 10.3389/fmicb.2016.00516

**Published:** 2016-04-18

**Authors:** Christopher E. Bagwell, Amanda Abernathy, Remy Barnwell, Charles E. Milliken, Peter A. Noble, Taraka Dale, Kevin R. Beauchesne, Peter D. R. Moeller

**Affiliations:** ^1^Environmental Sciences and Biotechnology, Savannah River National Laboratory, AikenSC, USA; ^2^Department of Biological Sciences, Alabama State University, MontgomeryAL, USA; ^3^Bioscience Division, Los Alamos National Laboratory, Los AlamosNM, USA; ^4^National Oceanic and Atmospheric Administration/National Centers for Coastal Ocean Science’s Center for Human Health Research Hollings Marine Laboratory, CharlestonSC, USA

**Keywords:** microalgae, bioactive metabolites, iron, crop protection, predation

## Abstract

Microalgae could become an important resource for addressing increasing global demand for food, energy, and commodities while helping to reduce atmospheric greenhouse gasses. Even though *Chlorophytes* are generally regarded safe for human consumption, there is still much we do not understand about the metabolic and biochemical potential of microscopic algae. The aim of this study was to evaluate biofuel candidate strains of *Chlorella* and *Scenedesmus* for the potential to produce bioactive metabolites when grown under nutrient depletion regimes intended to stimulate production of triacylglycerides. Strain specific combinations of macro- and micro-nutrient restricted growth media did stimulate neutral lipid accumulation by microalgal cultures. However, cultures that were restricted for iron consistently and reliably tested positive for cytotoxicity by *in vivo* bioassays. The addition of iron back to these cultures resulted in the disappearance of the bioactive components by LC/MS fingerprinting and loss of cytotoxicity by *in vivo* bioassay. Incomplete NMR characterization of the most abundant cytotoxic fractions suggested that small molecular weight peptides and glycosides could be responsible for *Chlorella* cytotoxicity. Experiments were conducted to determine if the bioactive metabolites induced by Fe-limitation in *Chlorella* sp. cultures would elicit protection against *Vampirovibrio chlorellavorus*, an obligate predator of *Chlorella.* Introduction of *V. chlorellavorus* resulted in a 72% decrease in algal biomass in the experimental controls after 7 days. Conversely, only slight losses of algal biomass were measured for the iron limited *Chlorella* cultures (0–9%). This study demonstrates a causal linkage between iron bioavailability and bioactive metabolite production in strains of *Chlorella* and *Scenedesmus.* Further study of this phenomenon could contribute to the development of new strategies to extend algal production cycles in open, outdoor systems while ensuring the protection of biomass from predatory losses.

## Introduction

Microscopic algae offer tremendous potential as a renewable source of clean burning liquid fuels, industrial chemicals, and high value commodities (Hannon et al., 2010; Sander and Murthy, 2010). Microalgae grow by capturing solar energy to power the conversion of carbon dioxide and inorganic nutrients into valued biochemicals, such as hydrocarbon substrates that can be readily converted to liquid transportation fuels. The sustainability and economic viability of large scale production of microalgal fuels and products, though, will require scientific and engineering advancements to improve our understanding of algal physiology, specifically carbon and energy flows to allow for controlled expression and maximum output of valued commodities (Brennan and Owende, 2010; Hannon et al., 2010; Fields et al., 2014).

Open, outdoor systems (i.e., ponds, raceways) are likely to be the most economical growth format for producing the vast quantities of algal biomass that would be required to meet the renewable fuels targets set forth by the U.S. Department of Energy [USDOE] (2010). Microalgal feedstocks will impose significant demands on nutrient and water resources for continuous operation (Pate et al., 2011; Bazilian et al., 2013). Site selection models are being applied to best match feedstock requirements to resource availability (Wigmosta et al., 2011; Venteris et al., 2014); for example, priority sites may permit access to eﬄuent streams from industry, agriculture, or wastewater treatment facilities to satisfy growth rate potentials. However, these resource inputs and the large surface area required for open pond designs make them highly susceptible to biological contamination. Biological contamination will have important impacts on the sustainability of algal production as well as the efficiency of downstream fuel conversion processes (Ugwu et al., 2008; Gao et al., 2012; Letcher et al., 2013; Smith and Crews, 2014). The lack of crop protection options and practical solutions to control biological contamination is a major obstacle in algal biofuels production.

Bacteria, cyanobacteria, and regional algae can be introduced into an open pond growth system via the water supply and/or resource streams, as well as by atmospheric deposition. These organisms will compete with the intended algal ‘crop’ for habitat, light, and nutrients. The potential for growth of nuisance strains or toxin producing species must also be considered as many cyanobacteria and microalgae are prolific producers of potent toxins and bioactive metabolites (Leflaive and Ten-Hage, 2007; Berry et al., 2008; Gademann and Portmann, 2008; Bertin et al., 2012a,b; de Morais et al., 2015) which could inadvertently impact the usage of harvested biomass for food or feed preparations, for example. Furthermore, viruses, fungi, and micro zooplankton grazers and predators can significantly and consistently reduce biomass and/or commodity yields (Letcher et al., 2013; Gong et al., 2015). Once established, herbivorous consumers can effectively destroy an algal crop in as little as a few days (Carney and Lane, 2014; Smith and Crews, 2014; Van Ginkel et al., 2015). Integrated pest management entailing selective application of chemical herbicides and pesticides has been successfully demonstrated (McBride et al., 2014; Xu et al., 2015), however, reliance on these treatments will increase operational costs and prolonged use could select for resistance in pest populations. Additional options that have been discussed in the literature include ecological engineering of aquatic communities to promote beneficial biological and/or chemical interactions (de-Bashan et al., 2004; Leão et al., 2010; Mendes and Vermelho, 2013; Bagwell et al., 2014; Carney and Lane, 2014; Kazamia et al., 2014; Smith and Crews, 2014), as well as the development of new biotechnologies to enable genetic and metabolic engineering for trait or strain development (Henley et al., 2013; Rasala et al., 2014). This area of research is receiving tremendous attention because the future success of algal derived fuels and commodities hinges on our ability to manage and control the biology of these engineered ecosystems.

Resource competition and predator – prey interactions play a pivotal role in shaping planktonic community composition and productivity, and these trophic dynamics that will likely be intensified in engineered systems intended for high density production of microalgae. Microalgae, though, have a variety of inducible defenses and adaptations that can be used to gain a competitive advantage or increase survivorship (Legrand et al., 2003; Pohnert et al., 2007; Granéli et al., 2008). For example, specific chemicals released by *Daphnia* during feeding induce colony formation in *Scenedesmus* spp.; these cell aggregates are too large to be consumed by the predator (Zhu et al., 2015). Other inducible defenses are chemically mediated, including the production of protective or deterrent secondary metabolites (i.e., allelochemicals), infochemicals, or toxins (Ianora et al., 2006). Allelopathy in planktonic systems has been known for some time and describes the production of bioactive metabolites by one organism to specifically influence the growth, survival, or reproduction of a target organism(s). Approximately 40 allelopathic species of microalgae have been described and the production of chemical defenses is enhanced by stress conditions; including nutrient limitation (most typically described for N and P), changes in pH and temperature, community composition and abundance, as well as grazing pressure (Wolfe, 2000; Legrand et al., 2003; Tillmann, 2003; Ianora et al., 2006; Granéli et al., 2008; Macías et al., 2008; Van Donk et al., 2011). Bioactive metabolite production by toxic algae is a compelling defensive strategy thought to inhibit the growth of competitors and deter grazers during a ‘bloom’ of rapid growth and high cell densities (Tillmann, 2004; Granéli et al., 2008). The production of allelochemicals by biofuel candidate strains of microalgae has not been systematically investigated, but the potential for allelopathic interactions to naturally influence, or to be used to intentionally control, the biotic structure of open pond systems is intriguing.

The aim of this study was to conduct a preliminary evaluation of selected biofuel candidate strains of unicellular green algae for innate predatory defenses that may hold promise for exploitation in the development of algal crop protection strategies. This investigation specifically examined the response of dense algal cultures to typical stress scenarios used to trigger triacylglycerides (TAG) biosynthesis (i.e., substrate for biofuel conversion) in algae. This study emphasizes a link between nutrient availability and bioactive metabolite production in microalgae, and while more work is needed to better understand the physiology and mechanisms involved; inducible defenses and allelochemicals should be examined as part of an overall strategy for achieving the true production potential of algae.

## Materials and Methods

### Strain and Culture Conditions

*Scenedesmus* sp. (strain 18B) and *Chlorella* sp. (strain 15) were obtained from a regional culture collection (Bagwell et al., 2014) and selected for this investigation based on prior performance in laboratory growth studies. The strains were grown to high density in nutrient replete M8 medium (10 L) with 18 h full spectrum white light/6 h dark cycling and continuous air bubbling. Once stationary phase was reached (as indicated by stabilization in total cell densities), biomass (∼5 g wet wt algae) was harvested by centrifugation (6,000 × *g*, 10 min, 15°C) and directly transferred to independent, 10 L glass column photobioreactors (custom built) containing modified growth medium devised to limit biomass for various macro- and/or micro-nutrients. Unmodified M8 medium is formulated to maximize biomass capacity and served as the nutrient replete experimental control (Mandalam and Palsson, 1998). Growth medium modifications (i.e., treatments) employed in this study are as follows. Treatment 1 was modified M8 medium having only ½ total PO_4_ (370 mg/L KH_2_PO_4_, 130 mg/L Na_2_HPO_4_^∗^2H_2_O) and a 1000x increase in Cu ∼0.1 mg/L CuSO_4_^∗^5H_2_O (calculated final concentration, 0.5 μM). Copper sulfate is a commonly used algaecide and it was reasoned that low level applications might lend additional stress to microalgae cultures during production. Treatment 2 was N8 medium which is purported to limit algal biomass for N, Mg, S, and Fe (Mandalam and Palsson, 1998). Treatment 3 was modified M8 medium which contained 1/100th KNO_3_ (30 mg/L). Treatment 4 was modified M8 medium which contained 1/10th total iron (1 mg/L Fe(III)-EDTA, 13 mg/L Fe(II)SO_4_^∗^7H_2_O), and Treatment 5 was also modified M8 medium which contained 1/100th total iron (0.1 mg/L Fe(III)-EDTA, 1.3 mg/L Fe(II)SO_4_^∗^7H_2_O). Cultures were grown for 3 weeks before being submitted for cytotoxicity screening. These medium formulations were selected for laboratory experimentation with high density microalgal cultures and thus, are not expected to be strain optimized or necessarily nutrient limiting for practical applications.

### Flow Cytometry

Samples of *Scenedesmus* sp. (strain 18B) and *Chlorella* sp. (strain 15) cultures were shipped overnight to Los Alamos National Laboratory (LANL) on blue ice for analysis by flow cytometry. Multi-parameter flow cytometry measurements were obtained using a BD Accuri^TM^ C6 flow cytometer fitted with a 96-well plate autosampler. Prior to sample analysis, instrumentation fluidics were calibrated to a 250 μl volume in a 96-well round bottom deep well plate. Algal samples were diluted in the appropriate medium designated for each treatment to ensure a count rate of 1,000–10,000 events/second. All samples were independently run three times, each in duplicate, thereby giving six analytical replicates per treatment. Counts were collected at a set event value of 10,000 on the slow fluidics setting.

Algae cells were gated based on a dot-plot of side light scatter versus forward light scatter. Chlorophyll autofluorescence per cell was determined in the same experiment by using the 488 nm excitation laser and a 670 nm long pass emission filter in the flow cytometer. The green fluorescence intensity (488 nm ex, 530/30 nm em) of each event was also measured on these samples. These data was used as the unstained, background fluorescence intensity, to be subtracted from the BODIPY^®^ (505/515) stained replicates (below).

Neutral lipid content, specifically TAG, for *Scenedesmus* and *Chlorella* samples was examined by flow cytometry, using the green fluorescent neutral lipid stain, BODIPY^®^ (505/515; Life Technologies, D-3921). Briefly, samples were diluted in triplicate in appropriate medium, as described above. Samples were stained by adding a working stock of 400 μM BODIPY^®^ in 50% DMSO to a final concentration of 22.6 μM BODIPY^®^. Stained samples were incubated at RT in the dark for at least 30 min and analyzed within 3 h. 10,000 count events were collected per well, on the slow fluidics setting. Green fluorescence intensity (488nm excitation, 530/30 nm emission) was measured for each event. The arithmetic mean of the fluorescence intensity for the stained samples was subtracted from the arithmetic mean of the fluorescence intensity of the unstained samples and plotted in GraphPad Prism.

### Fluorescence Microscopy

BODIPY^®^ (505/515) stained cultures (8 μl) were also individually examined using a Zeiss Axioplan epifluorescence microscope fitted with a Zeiss 100x objective lens. Images were captured using a Nikon D7000 digital camera and Nikon Camera Control Pro 2 software. Fluorescence was observed using a 450–490 nm excitation laser and a 520 nm long pass emission filter, permitting the collection of BODIPY^®^ (505/515) and chlorophyll fluorescence in the same image.

### Cytotoxicity Assay

Cell mass and production media were separated by centrifugation (6,000 × *g*, 15 min, 15°C) and samples (5 g wet weight algae and 1 L production medium/treatment) were lyophilized for cytotoxicity bioassays conducted at the NOAA laboratory in Charleston, SC, USA. Briefly, elutropic solvent extractions (dichloromethane, methanol, and water) were performed on every sample; yielding partitioned samples having corresponding differences in polarity. Partitioned samples were dried under a nitrogen gas stream and recovered in 100 μL of methanol as the carrier solvent for cytotoxicity assay against two immortalized mammalian cell lines in a high throughput process. Assays utilized the rat pituitary GH_4_ C1 cell line (ATCC CCL-82.2) and the mouse neuroblastoma Neuro 2A (N2A) cell line (ATCC CCL-131) in a conventional MTT (3-(4,5-Dimethylthiazol-2-yl)-2,5-diphenyltetrazolium bromide) colorimetric reaction to establish cytotoxicity of algal or produced water samples (Mosmann, 1983). Briefly, mammalian cells were transferred to a 96-well culture plate at a concentration of 2.8 × 10^3^ cells/mL (100 μl per well) and incubated at 37°C in 5% CO_2_ for at least 4 h before use in the MTT assay. Fractionated algal samples were added to sample wells in triplicate at 4, 2, 1 μl as to inform of cytotoxic compound potency or relative quantity. Methanol was used as a negative vehicle control and chloroform was used as a positive cytotoxic control (4 μl/well). All reaction wells received MTT (15 μL), and plates were then incubated for 24 h at 37°C in 5% CO_2_. Assays were stopped by adding 100 μl of a 0.01% HCl (v/v), 10% SDS (w/v) solution to the reaction wells and color formation was measured using a plate reader at 570 nm. Samples that assayed as ‘bioactive’ were subsequently fractionated by liquid chromatography and base level characterization of bioactive components (compound tagging) was performed by LC/MS. Bioactive methanol fractions were then loaded on a long glass column (1″id × 2.5′ long) containing 50 g of Amino packing (Sepra NH2 50 μm, 65A, Phenomenex). The packing was charged with 100% ethyl acetate (EtoAC) and the sample(s) was loaded in EtoAC. A series of elutions were performed with 100% methanol, 95% methanol/water, 90% methanol/water, 85% methanol/water. Fractions were collected separately, dried, and then tested for cytotoxicity as described above. The 90% methanol fraction was the most active and subsequently carried to the next step of semi-preparative HPLC purification.

### Liquid Chromatography/Mass Spectroscopy (LC/MS)

Bioactive fractions (100 μl; produced as described above) were semi-purified on a Waters^®^ HPLC system equipped with a Luna C18 (3 μm) column (Phenomenex Corporation, Torrance, CA, USA) by isothermal (35°C) gradient elution (97% H_2_O/3% Acetonitrile to 100% Acetonitrile) at a 1.0 mL/min flow rate with a photodiode array detector. Molecular mass determinations were made with a Waters^®^ 1525 system equipped with a 2767 sample manager and Micromass ZQ Mass Spectrometer. Spectra were analyzed using the MassLynx^TM^ software system.

### Nuclear Magnetic Resonance (NMR)

Semi-preparative HPLC purified bioactive components were structurally analyzed by nuclear spectroscopy using a 700 MHz NMR Spectrometer Bruker AVANCE^TM^ III HD equipped with a Bruker CryoProbe^TM^. One-dimensional proton (^1^H) and carbon (^13^C) experiments were conducted using the Bruker ZG NMR pulse sequence and the Bruker ZGDC pulse sequence, respectively. Spectral analyses utilized the TopSpin^TM^ software (Bruker) to structurally characterize the partitioned samples.

### Elemental Analysis of Microalgal Biomass

Microalgal biomass (1 g wet weight) was prepared for elemental analysis according to Bagwell et al. (2008) in order to remove loosely sorbed metals from extracellular matrices. Aqua regia dissolution was performed by digesting algal biomass with 3:1 (v:v) mixture of HCl and HNO_3_ for 30 min. Samples were diluted to 10 ml in deionized water and analyzed on an Agilent 730 ES Simultaneous Inductively Coupled Plasma – Atomic Emission Spectrometer (ICP-AES). Yttrium was used as an internal standard and instrument calibration was performed with a NIST traceable elemental standard.

### Statistical Analyses

Orthogonal transformation of the elements to their principal components (PC) was performed in JMP^®^ (SAS). Pearson correlation coefficient was used to measure the linear correlation between elements. Principal component analysis (PCA) was determined using the matrix of distances, D, and Euclidean distance. To investigate and visualize differences between the elements, the first two principal components (PC1 and PC2) of the distance matrix D were retained and a projection of each sample time was calculated onto the (PC1, PC2) plane as a bi-plot. Projecting sample time on the ordination plot reveals the relative contribution of time to the ordination of the elements.

### Predation Studies

*Vampirovibrio chlorellavorus* cultures were maintained as co-cultures with *Chlorella sorokiniana* (strain DOE 1412) in standard formulation BG-11 growth medium. Prior to experimentation, a working stock culture of *V. chlorellavorus* was prepared by recovering the aqueous phase (30 mL) of gravity settled (10 min) *C. sorokiniana* co-cultures. *V. chlorellavorus* cells were harvested by centrifugation (10,000 × *g* for 5 min) and the bacterial pellet was re-suspended in freshly prepared M8 medium (15 mL) containing no added iron. C*hlorella* sp. (strain 15) stock cultures were prepared under iron limited growth conditions corresponding to Treatments 4 and 5 as described above, and cultures grown in unmodified M8 medium were designated as controls. Cytotoxicity bioassays were performed on *Chlorella* sp. (strain 15) biomass harvested from all treatments, as described above, to confirm cytotoxicity for the Fe-limited cultures (Treatments 4 and 5) and no-reactivity for the control culture. Aliquots (24 mL) from each of the primary cultures (10^6^ cells/mL) were transferred in triplicate to sterile snap cap tubes with the predator *V. chlorellavorus* (1 mL) or without (1 mL sterile M8 medium). Experimental co-cultures were held at 28°C in the dark for 7 days. Viable algal cell counts that were determined microscopically (60× objective) using a hemocytometer.

## Results and Discussion

### Culture Screening for Cytotoxicity and TAG

**FIGURE 1:**
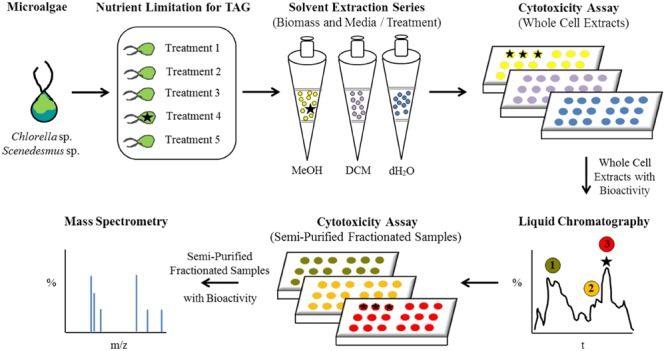
**Flow diagram illustrating the experimental design and analytical characterization of bioactive metabolites from microalgae**.

A conceptual illustration of the experimental design and analyses performed for bioactive metabolite induction, detection, and recovery from microalgae is summarized in **Figure 1**. *Scenedesmus* sp. (strain 18B) and *Chlorella* sp. (strain 15) cultures were grown in a variety of medium formulations intended to restrict biomass for macro- or micro-nutrients to simulate conditions that could be encountered during algal biomass production for biofuels or other high value commodities. Neutral lipid production, chiefly as TAG, is a well characterized response to nutrient limitation or environmental stress in diverse microalgae, including *Scenedesmus* and *Chlorella* strains, yet their ability to synthesize potentially beneficial bioactive metabolites in response to these stressors has not been investigated (e.g., Kellam et al., 1988). Interestingly, when iron was a limited nutrient, both strains yielded solvent fractionated samples that exhibited cytotoxicity when presented to mammalian cells (**Table 1**). Corresponding liquid chromatographs and total ion current plots for these solvent fractionated whole cell preparations are provided as Supplementary Figures S1–S6.

**Table 1:** Cytotoxicity demonstrated for freshwater green algae in response to nutrient limitation.

Strain	Revised medium formulation	Elutropic solvent series		
		DCM	MeOH	H_2_0
***Scenedesmus* sp. (Strain 18B)**	Treatment 1 (^1^/_2_ PO_4_, 1000x Cu^2+^)	–	–	–
	Treatment 2 (N8: Limited N, Mg, S, Fe)	–	+^N2A^	–
	Treatment 3 (^1^/_100_ NO_3_)	–	–	–
	Treatment 4 (^1^/_10_ total iron)	+^GH4C1/N2A^	++^GH4C1/N2A^	++^GH4/N2A^
	Treatment 5 (^1^/_100_ total iron)	ND	ND	ND
***Chlorella* sp. (Strain 15)**	Treatment 1 (^1^/_2_ PO_4_, 1000x Cu^2+^)	–	–	–
	Treatment 2 (N8: Limited N, Mg, S, Fe)	–	–	–
	Treatment 3 (^1^/_100_ NO_3_)	–	–	–
	Treatment 4 (^1^/_10_ total iron)	–	+++^GH4C1/N2A^	–
	Treatment 5 (^1^/_100_ total iron)	–	+++^GH4C1/N2A^	–

*The potency of algal cell extracts to the mammalian cells was inferred from the bioassay volume resulting in cell death; thus, 1 μl = +++, 2 μl = ++, and 4 μl = +. Cytotoxic activity is inferred from the immortalized mammalian cell lines GH4C1 and N2A which exhibit predominantly Ca^2+^ channel activity and Na^+^ channel activity, respectively. Bioassay results where no cytotoxicity was detected is indicated by ‘–’ and ‘ND’ indicates bioassays could not be performed because of poor growth and insufficient biomass.*

**FIGURE 2:**
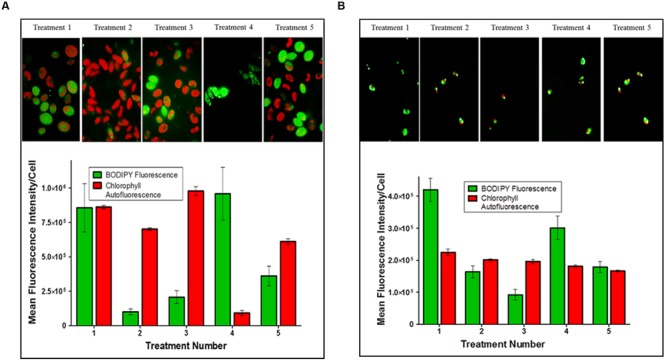
**triacylglycerides (TAG) induction in microalgal cultures.** The arithmetic mean and standard deviation (*n* = 6) in fluorescence intensity per cell for **(A)** *Scenedesmus* sp. (strain 18B) and **(B)** *Chlorella* sp. (strain 15) treatment populations analyzed by multi-parameter flow cytometry. Cells were stained with BODIPY^®^ 505/515 for TAG accumulation and auto-fluorescence was measured for chlorophyll content.

Plots of the mean fluorescence intensity for TAGs (BODIPY 505/515 staining) and chlorophyll autofluorescence of *Scenedesmus* sp. (strain 18B) cultures, as measured by flow cytometry, between treatments are shown in **Figure 2A**. Treatment 1 resulted in high cell to cell variability as fluorescence signals for TAG (BODIPY^®^) and chlorophyll were both equally high. Cultures from Treatments 2, 3, and 5 showed low TAG accumulation as indicated by BODIPY^®^ fluorescence relative to significantly higher chlorophyll content. Finally, Treatment 4 produced a highly chlorotic culture as indicated by the very low chlorophyll auto-fluorescence signal. The TAG induction response for Treatment 4, though, was strong and unanimous among cells in the culture, large lipid bodies were observed by microscopy.

Bioactivity was measured from all three extraction phases performed on *Scenedesmus* Treatment 4 biomass, though relative activity appeared to trend with solvent polarity (DCM < MeOH = H_2_O; **Table 1**). Biomass yields in Treatment 5 were too low to perform a valid cytotoxicity assay, no results are provided for this treatment. Cytotoxicity was also measured for *Scenedesmus* Treatment 2 biomass though the bioassay reaction was weak and inhibitory to only 1 of the mammalian cell lines.

Iron levels in N8 medium (Treatment 2) are comparable to those in Treatment 4 (10 mg/L and 14 mg/L, respectively), however, N8 medium is intended to also limit cell growth for N, Mg, and S (Mandalam and Palsson, 1998). *Scendesmus* sp. (strain 18B) was quite sensitive to iron limitation, compared to *Chlorella* sp. (strain 15) described below, suggesting possible differences between cultures in their physiological requirement for iron or their ability to efficiently transport iron into the cell. *Scenedesmus* cultures were quick to undergo chlorosis in response to iron limitation and growth yields were exceptionally low (<1 g/L wet weight); still, potent cytotoxic metabolites could be extracted. Because of the severe constraints in *Scenedesmus* biomass production in response to nutrient limitation, however, we could not pursue the *Scenedesmus* sp. (strain 18B) culture in any meaningful way. These results are significant, however, because *Scenedesmus* strains are being actively pursued for large scale production of biofuels and commodities (Fields et al., 2014; Hamed and Klöck, 2014; McBride et al., 2014), and this study provides an interesting starting point for future investigations of better performing strains that may also elicit production of bioactive metabolites under similar treatments.

*Chlorella* sp. (strain 15) cultures proved to be generally robust and insensitive to macro- or micro-nutrient limitation as assessed by growth yields, which typically exceeded 5 g/L (wet weight), and microscopic inspection by auto-fluorescence. Plots of the mean relative fluorescence (BODIPY^®^ and chlorophyll) of *Chlorella* cultures between treatments are shown in **Figure 2B**. Treatments 1 and 4 both induced TAG accumulation as indicated by BODIPY^®^ fluorescence in these cultures relative to the auto-fluorescence of chlorophyll. TAG induction in Treatment 1 was clearly the strongest, and while the measured BODIPY^®^ signal response for Treatment 4 was modest; relative to chlorophyll auto-fluoresce and by comparison to other treatments, we can conclude that iron depletion prescribed by this treatment did induce TAG accumulation. Conversely, high cell to cell variability measured for Treatments 2, 3, and 5 indicate physiological asynchrony among cells at different stages of growth and TAG accumulation. As performed, these treatments failed to produce a demonstrable TAG induction signal in *Chlorella* sp. (strain 15) as indicated by BODIPY^®^ fluorescence.

Cytotoxic activity was consistently high for the methanol soluble fractions obtained from *Chlorella* biomass harvested from Treatments 4 and 5 (**Table 1**). The strength of the cytotoxicity response was unaffected by a 4x dilution of the algal extracts, which we interpreted to signify a high abundance or potency (reactivity) of the bioactive metabolites present in those extracts.

The experimental design (i.e., medium formulations, growth/incubation time) was not optimized for maximum TAG production by these microalgal strains. Analysis by flow cytometry clearly showed a pronounced stress response imposed by a few treatments (*Scenedesmus* Treatment 4 and *Chlorella* Treatments 1 and 4) as reflected by BODIPY^®^ fluorescence for TAG relative to chlorophyll autofluorescence (**Figure 2**). All other treatments failed to impose a strong induction response for TAG biosynthesis within the time frame permitted for these experiments. Low BODIPY^®^ fluorescence relative to high or equal intensity chlorophyll autofluorescence implies these cultures had yet to fully transition out of photoautotrophic growth. Still, we are encouraged by the initial results that suggest the possibility of coordinating TAG biosynthesis and bioactive metabolite production in these microalgal strains.

The cytotoxicity outcomes were independently confirmed in three different growth studies. When both cultures were grown in iron depleted medium, we were able to consistently and reproducibly extract methanol soluble cytotoxic fractions from both strains. Based on these preliminary results, we hypothesize that either iron is an important trigger for induction of cytotoxic metabolite production in these green microalgae strains, or iron itself represents a critical chemical component whereby complexation with organic metabolites modulates compound reactivity (e.g., Oppenheimer et al., 1979; Gademann and Portmann, 2008). Iron has been implicated as important trigger for the production of chemical defenses in algae and dinoflagellates (Moeller et al., 2007; Gademann and Portmann, 2008; Bertin et al., 2012b); however, the inherent chemical and biological complexity of natural systems and the synergistic effects of nutrients has complicated mechanistic deduction of pathways that regulate cellular toxicity (e.g., Silva, 1990; Plumley, 1997; Larsen and Bryant, 1998; Kuffner and Paul, 2001; Anderson et al., 2002; Leflaive and Ten-Hage, 2007; Hardison et al., 2013).

### Correlation between Iron and Cytotoxicity

In order to test this hypothesis a series of experiments were performed in which iron was added back to *Chlorella* sp. (strain 15) cultures once cytotoxicity was established. If our supposition is correct, that iron depletion is a trigger for bioactive metabolite production, supplementation with new iron should reduce the cytotoxicity of the algal biomass in a time and/or concentration dependent manner. Again, *Chlorella* cultures (10 L) were limited for iron as prescribed in Treatment 4 (1/10 total iron) and Treatment 5 (1/100 total iron) to induce cytotoxicity (start of the experiment, *T* = 0), which was confirmed by *in vivo* bioassay and LC-MS fingerprinting of the methanol soluble cell fractions. Once cytotoxicity was confirmed, iron levels were fully restored for Treatment 4 *Chlorella* cultures; i.e., 0.1 mg/L Fe(III)-EDTA and 1.3 mg/L Fe(II)SO_4_ × 7H_2_O was added to the cultures. Iron-replete conditions were established for the Treatment 5 *Chlorella* cultures; i.e., 40 mg/L Fe(III)-EDTA and 520 mg/L Fe(II)SO_4_ × 7H_2_O was added to the cultures. Upon the addition of iron, deep green pigmentation was restored to cultures of both treatments, signifying a shift from a chlorotic state to chlorophyll biosynthesis.

**FIGURE 3:**
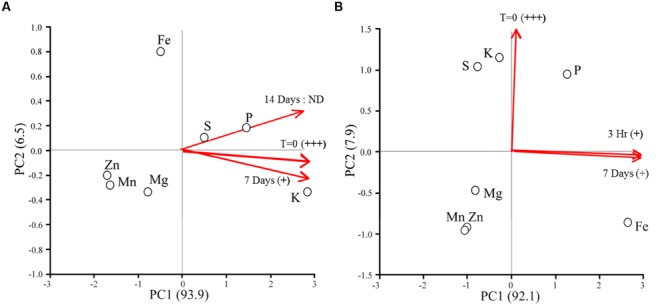
**Ordination plots of elements for cytotoxic *Chlorella* sp. (strain 15) cultures responding to the addition of new iron.** Red lines indicate the relative contribution of sampling time to the positioning of the elements in the ordination. Iron levels were fully restored for Treatment 4 *Chlorella* cultures **(A)**; i.e., 0.1 mg/L Fe(III)-EDTA and 1.3 mg/L Fe(II)SO_4_ × 7H_2_O was added to the cultures. Iron-replete conditions were established for the Treatment 5 *Chlorella* cultures **(B)**; i.e., 40 mg/L Fe(III)-EDTA and 520 mg/L Fe(II)SO_4_ × 7H_2_O was added to the cultures. Relative cytotoxic activity was inferred by *in vivo* bioassay and is denoted as highly active (+++), low level of activity (+), and no activity detected (ND).

**Figure 3** shows the elemental responses of *Chlorella* sp. (strain 15) cultures following the addition of iron as a function of time. Relative cytotoxicological activity responded in a time dependent manner in all cultures for both treatments following the addition to iron. Cytotoxicity in Treatment 4 (**Figure 3A**) cultures was just detectable after 7 days and no activity could be detected at 14 days. Interestingly, ordination plots of elements revealed that cellular contents of potassium (K), sulfur (S), and phosphorous (P) most strongly distinguished these time dependent samples, and that time had the greatest effect on S and P concentration, not K, at 14 days.

For the Treatment 5 cultures, only weak cytotoxicological activity could be measured at 7 days. Again, ordination plots suggest a time dependent response in elemental composition of microalgal cultures (**Figure 3B**). Samples taken at the start of the experiment, time = 0, were distinguished by sulfur (S) and potassium (K) concentration. Conversely, phosphorous (P) and iron (Fe) were more affected by time than the other elements included in the analysis, with Fe being the most responsive.

These results combined imply decreased potency (inactivation) or turnover of the cytotoxic metabolites in response to added iron. However, it remains unclear whether iron itself is the cue for bioactive metabolite production or turnover, or if the condition restricts cells for other nutrients (i.e., K, S, and P) which then triggers bioactive metabolite production. In fact, the production of allelochemicals and toxins by microalgae in response to co-limiting or unbalanced concentrations of nitrogen (N), phosphorous (P), and iron (Fe) are well documented (Granéli et al., 2008; Van Donk et al., 2011; Xu et al., 2013). Over the course of this experiment, algal cell numbers were statistically invariant (*p* = 0.33) between treatments, thus the changes in phenotype observed were due to a metabolic response(s) to available iron and were not an indirect consequence of changes in cell densities.

All living organisms require iron but this micronutrient is often in limited supply or chemically unavailable in many aquatic environments (Martin et al., 1994; Wilhelm, 1995; Hecky and Kilham, 1988; Boyd et al., 2007; Lis et al., 2015). In response to iron limitation many eubacteria, cyanobacteria, fungi, and algae will synthesize iron chelating metabolites, modulate virulence, and in some cases, release toxins or other bioactive metabolites to gain a competitive advantage for limited iron supplies (Loper and Buyer, 1991; Buysens et al., 1996; Mey et al., 2005; Paulsen et al., 2005; Gademann and Portmann, 2008; Ding et al., 2014). For example, *Anabaena flos-aquae* releases metal chelating siderophores as well as allelopathic chemicals to suppress the growth of competitors (Matz et al., 2004; Granéli et al., 2008). Additionally, the biosynthesis of authentic marine toxins domoic acid (produced by *Pseudo-nitzschia*; Mos, 2001) and microcystins (produced by *Microcystis aeruginosa*; Lukač and Aegerter, 1993) has been shown to be directly linked to iron bioavailability. To be clear, we are not suggesting that *Chlorella* and *Scenedesmus* are authentic toxin producers or that these compounds are necessarily hazardous. Many microalgae, including biofuel candidate strains, are known to synthesize bioactive metabolites and allelochemicals that act as a defense or a deterrent against competitors, predators and grazers (Tanaka et al., 1986; Ianora et al., 2006; Adolf et al., 2007; Leflaive and Ten-Hage, 2007; Gademann and Portmann, 2008; de Morais et al., 2015).

### Culture Susceptibility to Predation

Experiments were performed to determine if the bioactive metabolites induced by iron limitation in *Chlorella* sp. (strain 15) would elicit protection against a specific predator. *V. chlorellavorus* was selected for these experiments because this bacterium has been shown to be a particularly problematic threat in the outdoor cultivation of algae. This bacterium is an obligate predator of green algae with a host range restricted to *Chlorella* (Coder and Starr, 1978; Coder and Goff, 1986; Soo et al., 2015). Recently, *V. chlorellavorus* was identified in water samples collected during scaled up growth studies in outdoor greenhouses and raceways and most importantly, was determined to be responsible, or at least complicit, in crashes of *C. sorokiniana* cultures (Judith Brown, personal communication). The sequenced genome of *V. chlorellavorus* is consistent with an obligate predatory lifestyle which includes discrete phases of attachment to the *Chlorella* host, penetration into the host cell using a type IV secretion apparatus, consumption of leaked metabolites, cell division, and release (Soo et al., 2015).

**FIGURE 4:**
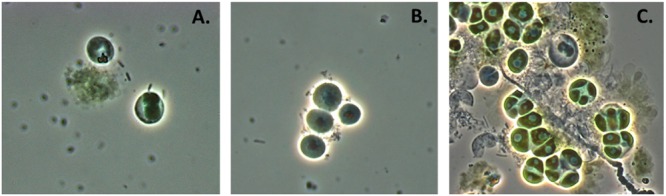
***Chlorella* culture under attack by *Vampirovibrio chlorellavorus.* (A)** Shows *V. chlorellavorus* (rods) seeking out and contacting algal cells. **(B)** Shows epibionts (coccoids) attached to host cells. **(C)** Shows the aftermath of lysed and degraded algal cells.

*Chlorella* biomass was prepared under iron limited conditions and methanol soluble fractions were assayed for cytotoxicity as described previously. Treatment 4 biomass (1/10 total iron), again, consistently generated highly bioactive preparations; fourfold dilution of the extract did not decrease cytotoxicity. Treatment 5 biomass, however, elicited a weak but detectable response only when bioassays were conducted with full strength (4 μl) preparations. Iron limited (cytotoxicity confirmed) and control *Chlorella* cultures were then incubated with the predator in the dark to suspend photoautotrophic growth of the host alga. Additionally, cultures were maintained in the depleted growth medium to preserve the physiological state (cytotoxic phenotype) of the host cultures for the duration of the experiment. Predation by *V. chlorellavorus* effectively destroyed the control cultures (**Figure 4**); viable cell counts were reduced on average by 72% in 7 days. Remarkably, no detectable cell loss was measured for Treatment 4 cultures (high level of relative cytotoxicity), and averaged viable cell loss for Treatment 5 cultures (low level of relative cytotoxicity) was only 9%. In a parallel experiment, iron was reintroduced by transferring the cytotoxic cultures to fresh M8 medium. Predatory losses for these cultures averaged 0 and 57% for Treatments 4 and 5, respectively. Standard deviation around the averaged cell counts did not exceed 15% for any of these experimental treatments. These results reinforce the postulated linkage between iron availability and the synthesis and potency of bioactive metabolites in this *Chlorella* strain. Importantly, the bioactive metabolites induced by iron limitation protected *Chlorella* biomass against epibiotic predation by *V. chlorellavorus*.

### Preliminary Characterization of Bioactive Metabolites

Approximately 450 g (wet weight) of viable *Chlorella* sp. (strain 15) biomass was produced under iron limiting conditions, as described for Treatment 4. Cytotoxic metabolites were captured by HPLC separation and fraction collection, and individual fractions were re-assayed for bioactivity (Supplementary Figures S5 and S6). Partial characterization of the most abundant bioactive sample by NMR provided spectra demonstrating the presence of diagnostic anomeric carbon resonances between 90 and 102 ppm (Supplementary Figure S7). These resonances coupled to oxygenated carbons found in the 68–85 ppm range are indicative of a glycosidic compound. It should be noted that these preparations for NMR were not of absolute purity and despite relatively high activity; chromatographic purification yielded only 50 μg of material which is insufficient for full structural characterization. For comparison, the complete characterization of the infochemicals released by grazing *Daphnia* which induce morphological defense in microalgae required 10 kg of starting material because the active compounds, aliphatic sulfates, were present in such low concentration (Yasumoto et al., 2008). In fact, the majority of allelopathic chemicals known to be produced by microalgae have not been formally characterized because of the technical limitations inherent to the study of molecules that are produced intermittently in response to unknown stimuli at sub - ng/L quantities (Legrand et al., 2003; Ianora et al., 2006; Leflaive and Ten-Hage, 2007; Granéli et al., 2008). Furthermore, several peaks observed by ^13^C NMR in the 50–70 ppm range could signify the presence of low molecular weight peptides. While the sugar signals were much stronger by comparison in these samples, we cannot definitively credit the observed activity strictly to a glycoside, nor can we discard the possibility of a synergistic mode of action.

## Conclusion

It is now evident that chemical signals play an important role in regulating phytoplankton community structure and trophic interactions (Legrand et al., 2003; Ianora et al., 2006, 2011; Pohnert et al., 2007; Van Donk et al., 2011). A better understanding of the ecological roles of potent allelochemicals and bioactive metabolites could help inform the development of new strategies to improve microalgal domestication and cultivation (Mendes and Vermelho, 2013). At this point we cannot meaningfully extrapolate these laboratory results to possible ecological consequences in a natural or engineered setting. While we have demonstrated the production of bioactive metabolites for two biofuel candidate strains of algae in response to iron limitation, the possibility that these metabolites function allelopathically or as infochemicals was not investigated here but should be considered in the future. The experimental treatments imposed here are relevant to the large scale production of algae because nutrient depletion will be inherent to dense algal cultures, or used as an operational strategy to cut cost or stimulate oil production for biofuels. Our results suggest that iron might be useful to help boost the production of neutral lipids (specifically TAG) in *Chlorella* and *Scenedesmus* if the timing of these events can be coordinated, as well as trigger the induction of a chemical defense against a particularly devastating bacterial predator, *V. chlorellavorus*. Further work is needed to evaluate the complex interactions between key environmental parameters, the expression and stability of bioactive metabolites *in vivo*, and to examine the possible ecological interactions mediated by these chemicals within planktonic communities. Inducible chemical defenses could help facilitate the development of new crop protection strategies for algal cultivation and production facilities.

## Author Contributions

CB, AA, RB, CM, PN, TD, KB, and PM contributed intellectual input and assistance to this study and manuscript preparation. CB developed the original framework. TD, KB, and PM contributed reagents and data analysis; CB, PN, and TD performed statistical analysis and data integration and CB wrote the paper.

## Conflict of Interest Statement

The authors declare that the research was conducted in the absence of any commercial or financial relationships that could be construed as a potential conflict of interest.
