# Supplementary material for: Discovery of Bioactive Metabolites in Biofuel Microalgae That Offer Protection against Predatory Bacteria
Source: Front Microbiol. 2016 Apr 18;7:516. doi: 10.3389/fmicb.2016.00516 (PMC4834574; doi:10.3389/fmicb.2016.00516)
Supplement: Supplementary file 1 [file Data_Sheet_1.DOCX]

Supplementary Material

Discovery of Bioactive Metabolites in Biofuel Microalgae that offer Protection against Predatory Bacteria

Christopher E. Bagwell*, Amanda Abernathy, Remy Barnwell, Charles E. Milliken, Peter A. Noble, Taraka Dale, Kevin R. Beauchesne, and Peter D.R. Moeller.

*** Correspondence:** [Christopher.bagwell@srnl.doe.gov](mailto:Christopher.bagwell@srnl.doe.gov)


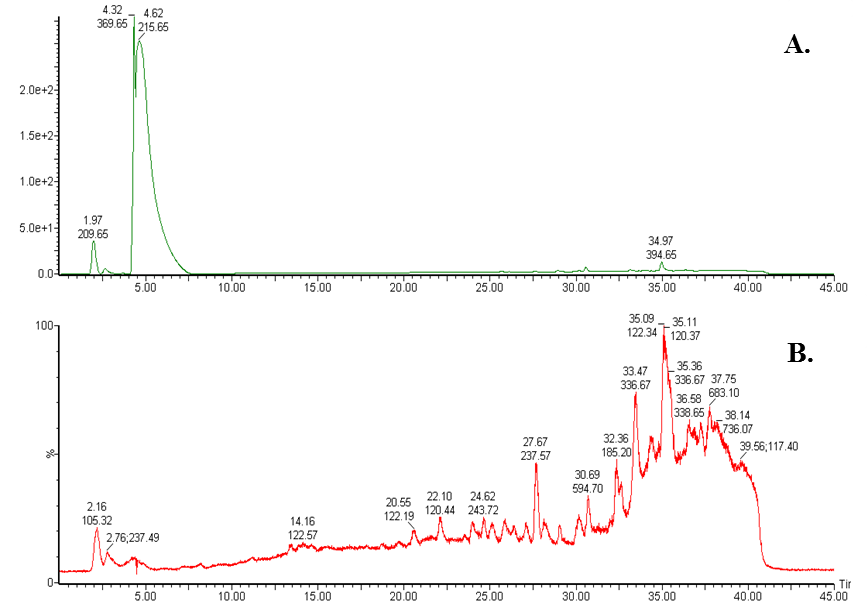


**Supplementary Figure 1.** Liquid chromatography (panel A) and total ion current plot (panel B) of bioactive methanol (MeOH) fractionated *Scenedesmus* sp. (Strain 18B) Treatment 2.

**
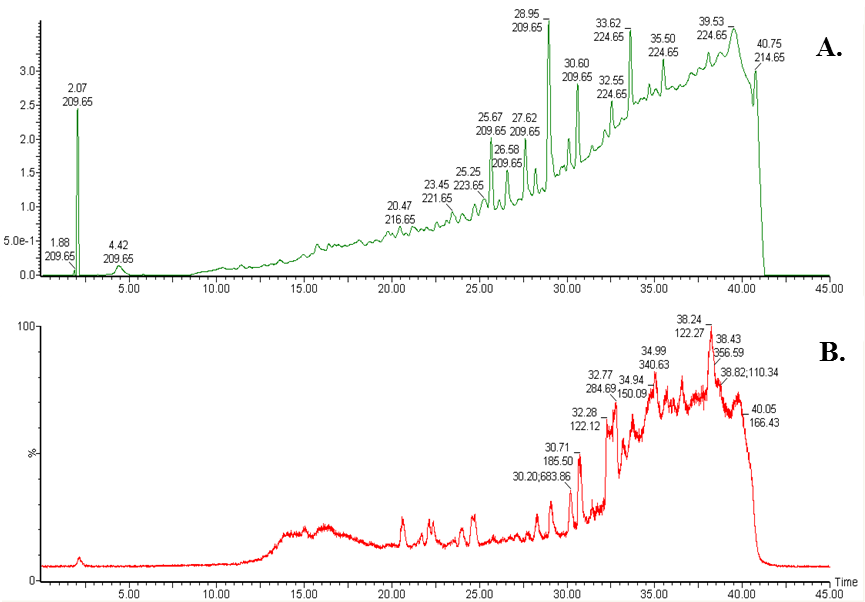
**

**Supplementary Figure 2.** Liquid chromatography (panel A) and total ion current plot (panel B) of bioactive dichloromethane (DCM) fractionated *Scenedesmus* sp. (Strain 18B) Treatment 4.

**
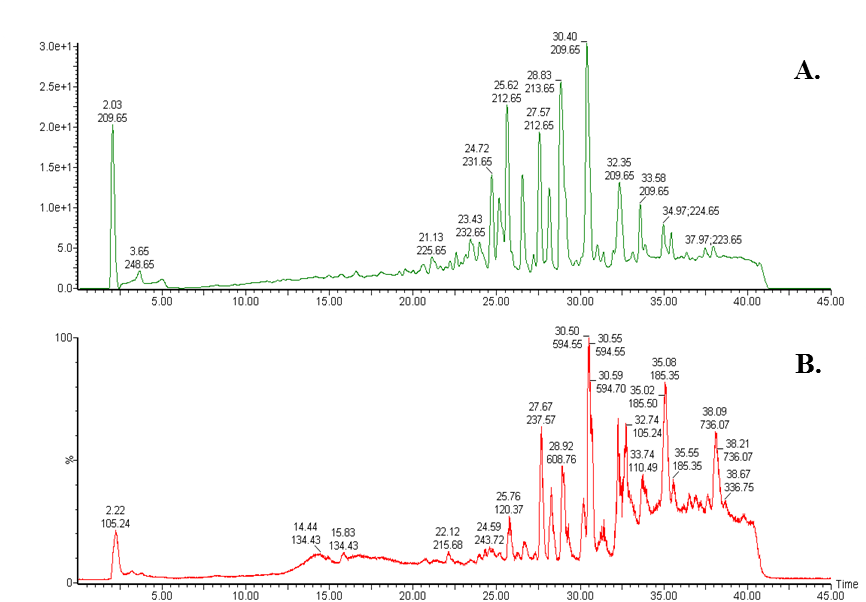
**

**Supplementary Figure 3.** Liquid chromatography (panel A) and total ion current plot (panel B) of bioactive methanol (MeOH) fractionated *Scenedesmus* sp. (Strain 18B) Treatment 4.


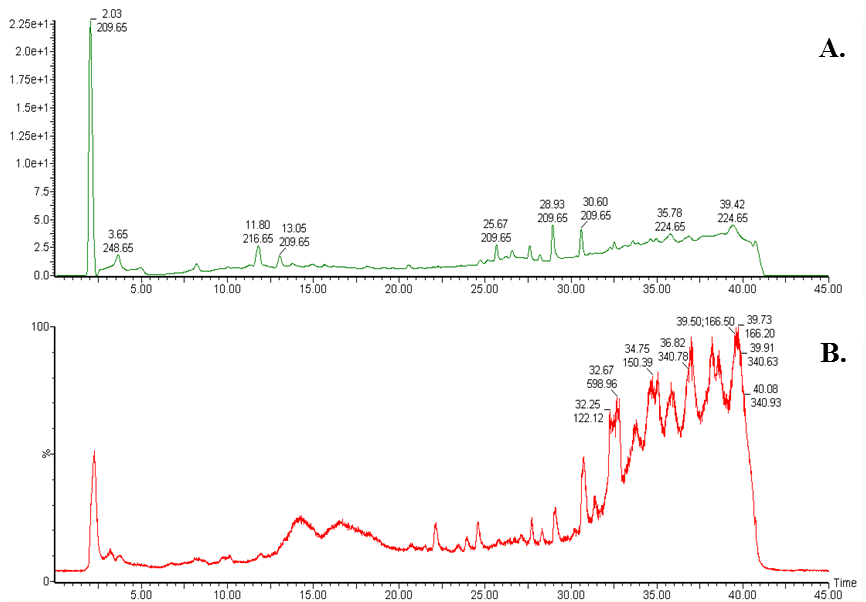


**Supplementary Figure 4.** Liquid chromatography (panel A) and total ion current plot (panel B) of bioactive water (H_2_O) fractionated *Scenedesmus* sp. (Strain 18B) Treatment 4.


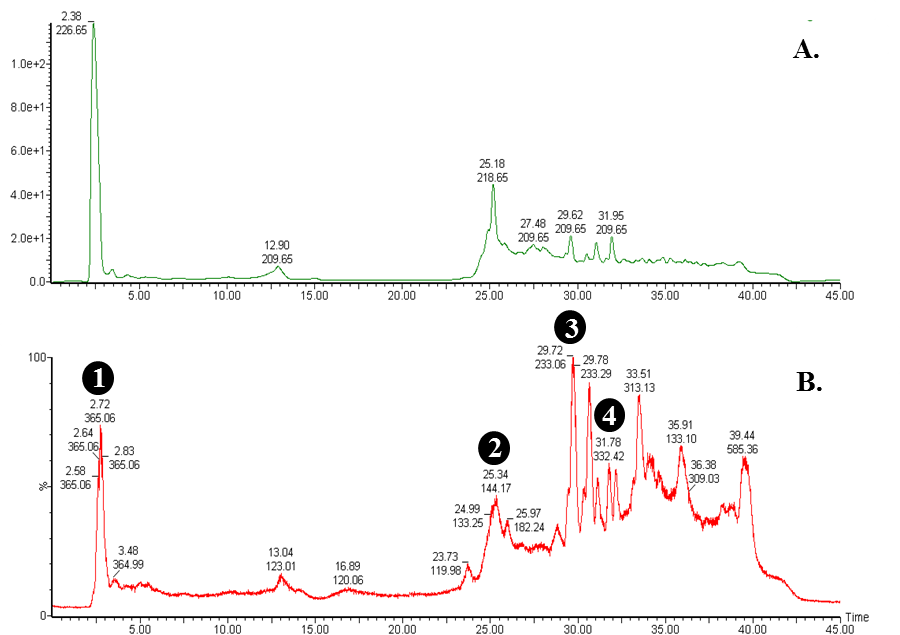


**Supplementary Figure 5.** Semi-preparative LC purification and total ion plots of bioactive methanol (MeOH) fractionated *Chlorella* sp. (Strain 15) Treatment 4.


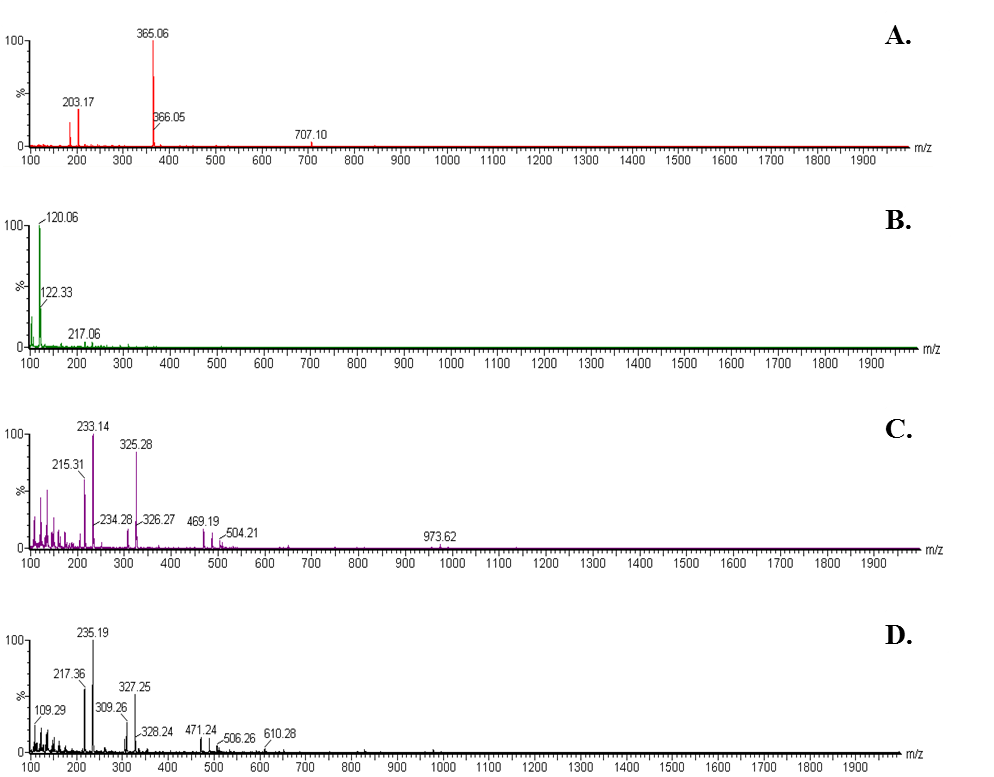


**Supplementary Figure 6.** Mass spectra of semi-preparative LC purified bioactive methanol (MeOH) fractionated *Chlorella* sp. (Strain 15) Treatment 4. The peaks labeled 1, 2, 3, and 4 in Supplementary Figure 5 correspond to panels A, B, C, and D, respectively.

**
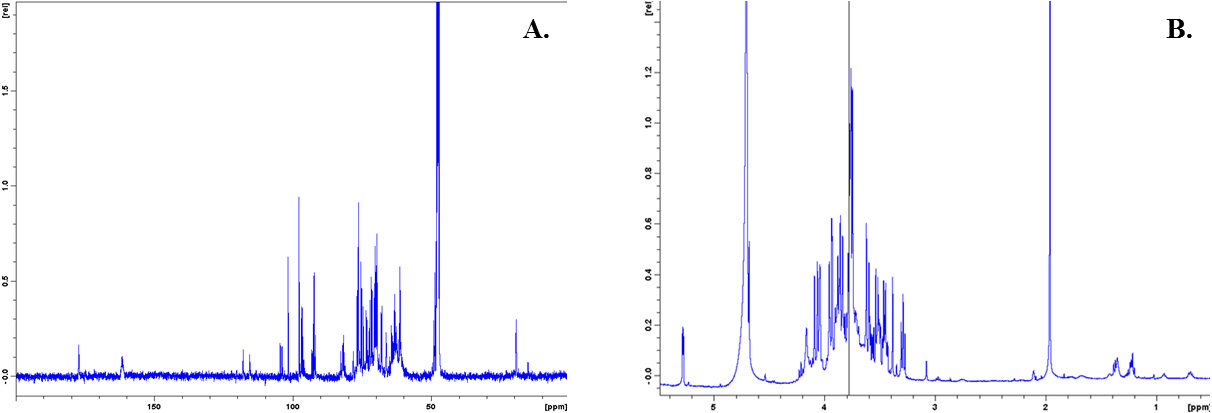
**

**Supplementary Figure 7.** ^13^C (panel A) and ^1^H (panel B) NMR spectra of a semi-preparative LC purified bioactive compound (Panel A, Supplementary Figure 6) obtained from methanol (MeOH) fractionated *Chlorella* sp. (strain 15) Treatment 4. In panel A, the anomeric carbon of the sugar group resonates at 90 – 102 ppm, and the region from 68 – 85 ppm reveals hydroxylated carbons typical of a sugar. The ^1^H spectra in panel B shows multiplets in the region of 3 – 4 ppm that are typical for protons found on carbons bound to the hydroxyl groups noted above. The spectra combined are indicative of a sugar joined to an uncharacterized functional group by a glycosidic bond.
